# Spontaneous synthesis of gold nanoparticles on gum arabic-modified iron oxide nanoparticles as a magnetically recoverable nanocatalyst

**DOI:** 10.1186/1556-276X-7-317

**Published:** 2012-06-19

**Authors:** Chien-Chen Wu, Dong-Hwang Chen

**Affiliations:** 1 Department of Chemical Engineering, National Cheng Kung University, Tainan, Taiwan, 701, Republic of China

**Keywords:** Gold nanoparticles, Spontaneous green synthesis, Magnetically recoverable catalyst, Nitrophenol, Catalytic reduction

## Abstract

A novel magnetically recoverable Au nanocatalyst was fabricated by spontaneous green synthesis of Au nanoparticles on the surface of gum arabic-modified Fe_3_O_4_ nanoparticles. A layer of Au nanoparticles with thickness of about 2 nm was deposited on the surface of gum arabic-modified Fe_3_O_4_ nanoparticles, because gum arabic acted as a reducing agent and a stabilizing agent simultaneously. The resultant magnetically recoverable Au nanocatalyst exhibited good catalytic activity for the reduction of 4-nitrophenol with sodium borohydride. The rate constants evaluated in terms of pseudo-first-order kinetic model increased with increase in the amount of Au nanocatalyst or decrease in the initial concentration of 4-nitrophenol. The kinetic data suggested that this catalytic reaction was diffusion-controlled, owing to the presence of gum arabic layer. In addition, this nanocatalyst exhibited good stability. Its activity had no significant decrease after five recycles. This work is useful for the development and application of magnetically recoverable Au nanocatalyst on the basis of green chemistry principles.

## Background

Nanoparticles have been widely investigated in the fields of chemistry, physics, electronics, biology, and medicine due to their unique physical and chemical characteristics which are different from bulk materials [1,2]. Among these researches, magnetic and noble metal nanoparticles (particularly Fe_3_O_4_ and Au) attracted considerable attention in the past decades. Magnetic nanoparticles have been extensively utilized in the recovery of metal ions and dyes, magnetic bioseparation, targeted therapy, drug delivery, and biological detection and imaging because magnetic separation technique possesses the advantages of rapidity, high efficiency, and cost-effectiveness [3-7]. Also, they have been shown to be highly efficient as supports in heterogeneous catalytic reactions owing to their high specific surface area and magnetically recoverability [8]. On the other hand, Au nanoparticles not only exhibit unique optical and catalytic properties but also have excellent chemical stability and biocompatibility. These characteristics lead to many potential applications such as in optics, electrochemistry, catalysis, and biochemical sensing [9-12].

The composite nanoparticles of Fe_3_O_4_ and Au combine the functions of these two components and can be applied in the waste water treatment. As has been reported, some noble metal nanoparticles stabilized with surfactant or dendrimer are capable for catalytic reduction of aromatic nitro compounds or dyes [13-15] because they have higher Fermi potential and can be used as catalyst for many electron-transfer reactions [16,17]. However, their recovery from such stabilizers-containing systems is not easy. To overcome this problem, using magnetic nanoparticles as their support is a superior strategy which makes them easy to recover. Thus, the composite nanoparticles of Fe_3_O_4_ and Au are expected to have great potential as magnetically recoverable catalyst for the treatment of waste water.

Various approaches for the synthesis of Fe_3_O_4_-Au composite nanoparticles have been reported, including the formation of Au shells on the polyelectrolyte modified Fe_3_O_4_ nanoparticles [18], reduction of Au ions on Fe_3_O_4_ nanoparticles with sodium citrate [19], electrostatic attraction of as-prepared Au nanoparticles onto Fe_3_O_4_/polypyrrole nanocomposites [20], thermal decomposition of Au(OOCCH_3_)_3_ on poly(vinylpyrrolidone)-modified Fe_3_O_4_ particles [21], and others [22,23]. However, it is inevitable that most of these processes involved the use of surfactants and reducing agents which are usually harmful for the environment and ecosystem.

In the past decade, the green chemistry which aims to reduce or eliminate hazardous substances in the design, development, and implementation of chemical processes and products is becoming more and more important [24,25]. So a lot of routes obeying the green chemistry principles have been developed for the synthesis of Au nanostructures by replacing toxic chemicals with environment-friendly materials [26-30]. Gum arabic (GA) is a polysaccharide which consists plenty of amino acids and hydroxyl groups on the polymer chains. It is known that these functional groups could reduce metal ions to metal nanoparticles through the oxidation mechanism [31,32]. Recently, we synthesized Au nanoparticles successfully by using GA as both reducing and stabilizing agent in the absence of other additives [33]. In our earlier work, we have also modified the surface of Fe_3_O_4_ nanoparticles with GA as a novel magnetic nano-adsorbent for the removal of heavy metal ions. Thus, it seems interesting and meaningful to fabricate the Au-Fe_3_O_4_ composite nanoparticles by the *in situ* green synthesis of Au nanoparticles on the GA-modified Fe_3_O_4_ nanoparticles. So, in this work, we proposed a new green route to fabricate Au-Fe_3_O_4_ composite nanoparticles by *in situ* synthesis of Au nanoparticles on GA-modified Fe_3_O_4_ nanoparticles. Furthermore, it is known that nitrophenols are the typical aromatic nitro pollutants in industrial and agricultural wastewaters. A lot of methods have been developed for their removal, including adsorption [34], microbial degradation [35], photocatalytic degradation [36], microwave-assisted catalytic oxidation [37], electro-Fenton method [38], electrocoagulation [39], and electrochemical treatment [40]. Because Au nanoparticles can serve as the electron relay between 4-nitrophenolate ion (oxidant) and BH_4_^−^ (reductant) for the catalytic reduction of 4-nitrophenol (4-NP) with sodium borohydride [41,42], the catalytic capability and performance of the resultant Au-Fe_3_O_4_ composite nanoparticles were demonstrated by investigating the catalytic reduction of 4-NP with sodium borohydride.

## Methods

Ferric chloride, 6-hydrate was purchased from J. T. Baker Chemical Company (Phillipsburg, NJ, USA). Ferrous chloride tetrahydrate, gum arabic, and sodium borohydride were obtained from Fluka (Fluka Chemical Corporation, Buchs, Switzerland). Ammonium hydroxide (29.6%) was supplied by TEDIA Company (Fairfield, OH, USA). Hydrogen tetrachloroaurate and 4-nitrophenol were purchased from Alfa Aesar (Ward Hill, MA, USA). All chemicals were of guaranteed or analytical grade reagents, commercially available, and used without further purification. The water used throughout this work was the reagent-grade water produced by Milli-Q SP ultra-pure-water purification system of Nihon Millipore Ltd., Tokyo, Japan.

GA-modified magnetic nanoparticles (GA-MNP) were prepared according to our previous work [8]. Firstly, iron oxide magnetic nanoparticles (MNP) were prepared by coprecipitation of Fe^2+^ and Fe^3+^ ions with ammonia solution and then followed by thermal treatment. The ferric and ferrous chlorides (with molar ratio of 2:1) were dissolved in water at a concentration of 0.3 M iron ions. Chemical precipitation was achieved by adding NH_4_OH solution (29.6%) at 25°C under vigorous stirring. During the reaction process, the pH was maintained at about 10. The precipitates were heated at 80°C for 30 min, then washed several times with water and ethanol, and finally dried in a vacuum oven at 70°C. Secondly, for the surface modification with GA, 100 mL of GA solution (10 mg/mL) was mixed with 1.0 g of MNP in a stoppered bottle. The reaction mixture was sonicated for 20 min, then mixed on a vortex mixer for 5 min, and was sonicated again for another 10 min. The product GA-MNP were recovered magnetically from the reaction mixture by using a permanent magnet with a surface magnetization of 6,000 G, then washed three times with 100 mL of distilled water, and finally dried in an air oven at 50°C for 24 h and stored in a stoppered bottle for further use.

For *in situ* green synthesis of Au nanoparticles on GA-MNP, hydrogen tetrachloroaurate (0.3 mM) was dissolved in an aqueous solution of GA-MNP (1 mg/mL) at first. Then, the solution was stirred gently at 55°C for 8 h to yield Au nanoparticles. The Au nanoparticles-loaded GA-MNP (GAAu-MNP) were recovered magnetically by a permanent magnet, then washed three times with distilled water, and finally dried in a vacuum chamber at room temperature. Since it was found that all Au(III) ions were reduced completely, the loading of Au nanoparticles on GA-MNP could be calculated to be 0.059 mg/g. The ultraviolet–visible (UV–VIS) absorption spectra of the resultant colloid solutions were monitored by a JASCO model V-570 ultraviolet–visible near-infrared spectrophotometer (JASCO Inc., Easton, MD, USA). The particle size was determined by transmission electron microscopy (TEM) on a Hitachi model HF-2000 field emission transmission electron microscope with a resolution of 0.1 nm. The high-resolution TEM image was observed by a JEOL model JEM-2100 F electron microscope (JEOL Ltd., Tokyo, Japan) operated at 200 kV. The samples for TEM analysis were obtained by depositing a drop of colloid solution onto a 200-mesh Formvar-covered copper grid and evaporating in a vacuum chamber at room temperature. An X-ray diffraction (XRD) measurement was performed on a Shimadzu model RX-III X-ray diffractometer (Shimadzu Corporation, Canby, OR, USA) at 40 kV and 30 mA with CuKα radiation (λ = 0.1542 nm). The samples for XRD analysis were washed twice with water, collected by centrifugation, and dried in a vacuum chamber overnight.

For the catalytic reduction of 4-NP with NaBH_4_, typically, 95 mL of aqueous solution containing NaBH_4_ and 4-NP was prepared at first. Then, 5 mL of aqueous solution containing GAAu-MNP (5 mg of GA-MNP and 0.295 mg of Au) was added to start the reaction. The yellow color of the solution gradually vanished, indicating the reduction of 4-NP. The variation of 4-NP concentration with time was monitored spectrophotometrically at a wavelength of 400 nm. The reaction temperature was controlled at 30°C. Also, the initial concentration ratio of NaBH_4_ to 4-NP was fixed at 100 so that the concentration of NaBH_4_ could be considered as a constant during the reaction. After the reaction, GAAu-MNP were collected by using a permanent magnet, washed two times with deionized water, and then reused for recycling in the experiment to examine the reusability.

## Results and discussion

**Figure 1:**
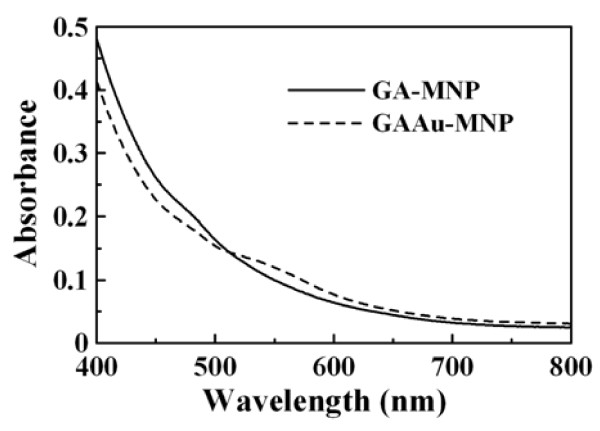
UV–VIS absorption spectra of GA-MNP and GAAu-MNP solutions.

Figure 1 shows the UV–VIS absorption spectra of GA-MNP and GAAu-MNP solutions. It was found that, as compared to GA-MNP, GAAu-MNP exhibited a characteristic absorption band of Au nanoparticles between 500 and 600 nm owing to the surface plasmon resonance. This demonstrated that Au nanoparticles could be successfully deposited on GA-MNP via the *in situ* reduction of Au(III) ions with GA. The characteristic absorption band of Au nanoparticles on GAAu-MNP was red-shifted and broader than that of GA-stabilized Au nanoparticles. This might be caused by the high sensitivity of metal nanoparticles to the surrounding environment [19].

**Figure 2:**
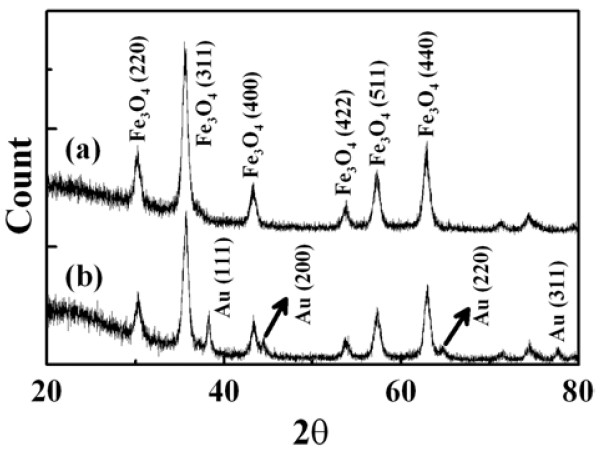
XRD patterns of GA-MNP and GAAu-MNP.

Figure 2 shows the XRD patterns of GA-MNP and GAAu-MNP. For GA-MNP, six characteristic peaks corresponding to the (220), (311), (400), (422), (511), and (440) planes of Fe_3_O_4_ were observed at 2θ = 30.1, 35.5, 43.1, 53.4, 57.0, and 62.6°, respectively. This confirmed that GA-MNP were composed of pure magnetite with a spinal crystal structure, [21] and the modification process did not result in the phase change of MNP. For GAAu-MNP, an additional characteristic peak at 38.1° and three weak peaks at 44.3, 64.5, and 77.4° were observed. They could be referred to the (111), (200), (220), and (311) planes of face centered cubic (fcc) Au, respectively, confirming the spontaneous formation of Au nanoparticles on GA-MNP.

**Figure 3:**
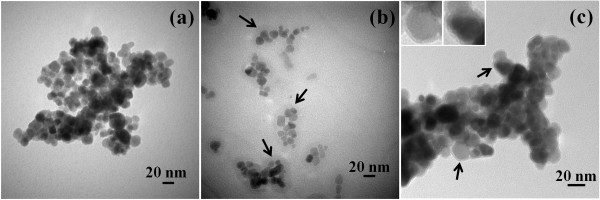
**Typical TEM images of MNP (a), GA-MNP (b), and GAAu-MNP (c).** The left-top insets in (c) are zoom-in images marked by arrows.

**Figure 4:**
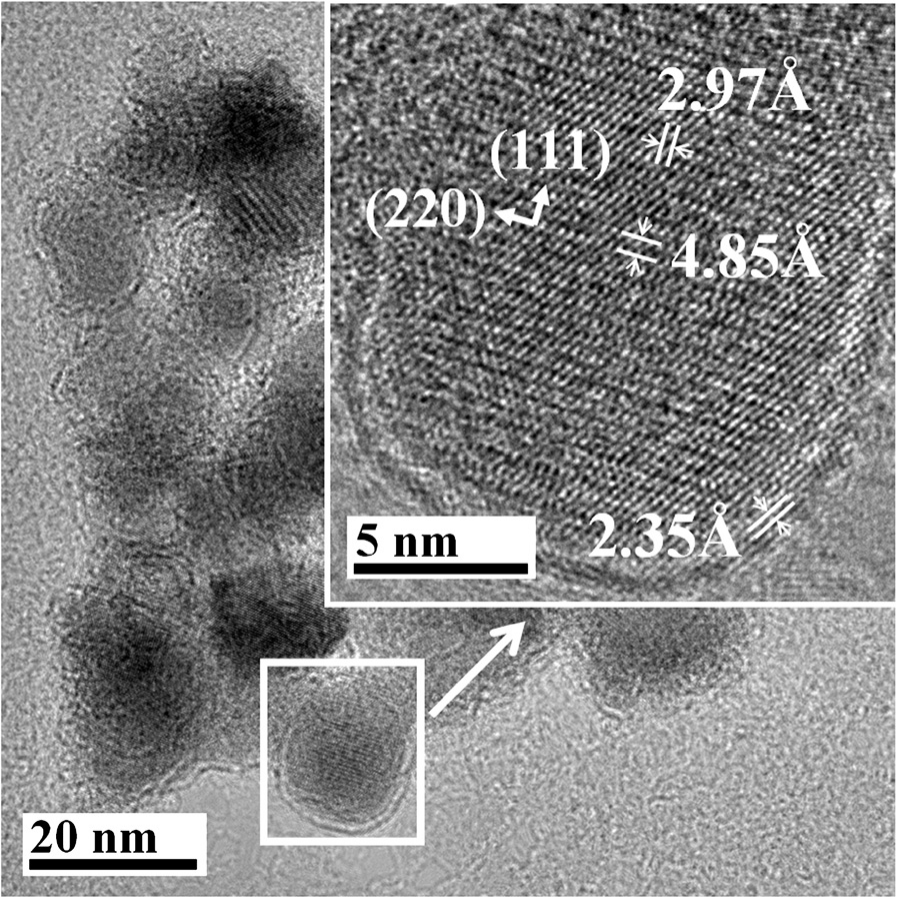
A typical high-resolution TEM image of GAAu-MNP.

Figure 3 shows the TEM images of MNP, GA-MNP, and GAAu-MNP. As indicated in Figure 3a, the MNP had a mean diameter of 17.4 ± 3.5 nm and were agglomerated to larger clusters slightly due to the magnetic force among them. After modification with GA, the resultant GA-MNP became discrete with a multi-cored structure as shown in Figure 3b. The formation of multi-cored structure could be resulted by the absorbed GA molecules which could absorb several adjacent MNP [43,44]. For the TEM image of GAAu-MNP as shown in Figure 3c, it was found that GA-MNP seemed to be covered by a layer of Au nanoparticles. To obtain a clearer image, its high-resolution TEM was shown in Figure 4. In the inner part of the composite particle, the crystalline nature of MNP with a clearly resolved lattice spacing of 4.85 and 2.97 Å could be observed. They are related to the (111) and (220) planes of spinel magnetite, respectively. Furthermore, the outer layer revealed that the Au shell had a thickness of about 2 nm and a lattice spacing of 2.35 Å, which was corresponding to the (111) plane of fcc Au.

**Figure 5:**
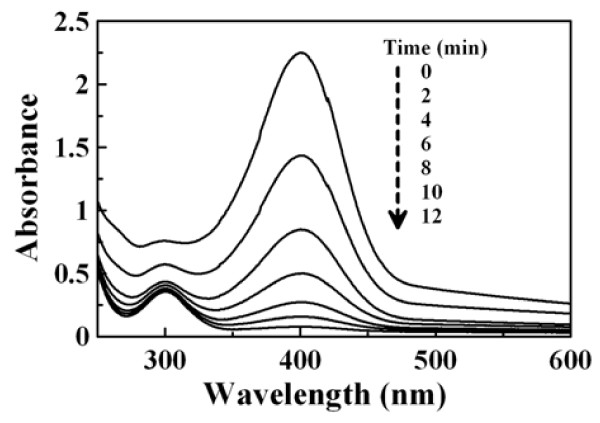
**Time-dependent UV/VIS absorption spectra for NaBH**_**4**_**-reduction of 4-NP catalyzed by GAAu-MNP.** [Au]_0_ = 0.295 × 10^−2^ mg/mL, [4-NP] = 1.5 × 10^−4^ M, [NaBH_4_] = 1.5 × 10^−2^ M, 30°C.

**Figure 6:**
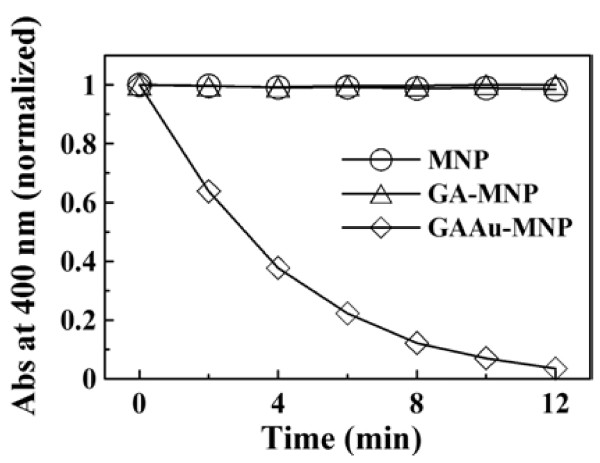
**Absorbance variation with time at 400 nm for NaBH**_**4**_**-reduction of 4-NP with different nanoparticles.** [Au]_0_ = 0.295 × 10^−2^ mg/mL, [4-NP] = 1.5 × 10^−4^ M, [NaBH_4_] = 1.5 × 10^−2^ M, 30°C.

The catalytic ability of GAAu-MNP was demonstrated by investigating the reduction of 4-NP with NaBH_4_. It is well known that 4-NP has a characteristic absorption peak at 317 nm. The addition of NaBH_4_ would cause the formation of 4-nitrophenolate ions and lead to the shift of characteristic absorption peak from 317 to 400 nm [45]. Also, in the presence of Au catalyst, 4-nitrophenolate ions could be further reduced to 4-aminophenol (4-AP) which exhibited a characteristic absorption peak at 300 nm. In this work, the preliminary experiment revealed that the yellow color of 4-NP solution became deeper after the addition of NaBH_4_, and a red shift of characteristic absorption peak from 317 to 400 nm occurred, confirming the formation of 4-nitrophenolate ions in the alkaline solution [46]. The absorbance remained unchanged at 400 nm even after several days, revealing that no reduction of 4-NP had occurred. However, in the presence of GAAu-MNP, the yellow color of 4-NP solution became colorless, finally owing to the reduction of 4-NP to 4-AP with NaBH_4_. As indicated in Figure 5, the time-dependent UV–VIS absorption spectra revealed that the addition of GAAu-MNP induced the fading of peak intensity at 400 nm and the appearance of a new absorption peak at 300 nm. This revealed the formation of 4-AP and demonstrated the catalytic ability of GAAu-MNP for the reduction of 4-NP with NaBH_4_. The decrease in the absorbance at 400 nm with time was shown in Figure 6. For comparison, the catalytic abilities of MNP and GA-MNP were also tested as indicated in Figure 6. It was found that the absorbance of 4-NP solution with NaBH_4_ at 400 nm remained unaltered after the addition of MNP or GA-MNP, revealing no significant reduction of 4-NP. This confirmed that the catalytic ability of GAAu-MNP for the reduction of 4-NP to 4-AP resulted from the Au nanoparticles deposited on GA-MNP.

**Figure 7:**
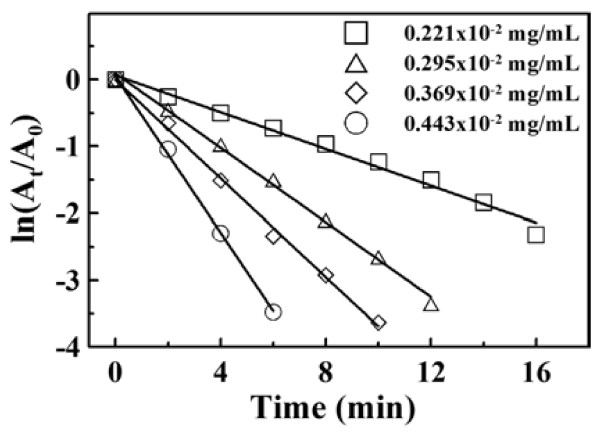
**Plots of ln(*A***_***t***_**/*A***_**0**_**) versus time for NaBH**_**4**_**-reduction of 4-NP at different GAAu-MNP amounts.** [4-NP] = 1.5 × 10^−4^ M, [NaBH_4_] = 1.5 × 10^−2^ M, 30°C.

Since 4-NP was present in the form of 4-nitrophenolate, the variation of the absorbance at 400 nm with time could be considered as the variation of 4-NP concentration with time. Therefore, the ratio of 4-NP concentration at time *t* to that at *t* = 0 could be directly given by their absorbance ratio *A*_*t*_/*A*_0_ [30,33 − 35]. Figure 7 shows the plots of ln(*A*_*t*_*/A*_0_) versus time at different amounts of GAAu-MNP. It was obvious that the reduction rate increased with the increase in the amount of GAAu-MNP. Also, the good linear correlations revealed that the NaBH_4_-reduction of 4-NP catalyzed by GAAu-MNP followed the pseudo-first-order kinetics. The corresponding pseudo-first-order rate constants (*k*) and correlation coefficients were determined as listed in Table 1. It was obvious that the pseudo-first-order rate constants increased with increasing the amount of GAAu-MNP.

**Table 1:** **Pseudo-first-order rate constants for the reduction of 4-NP with NaBH**_**4**_**in the presence of GAAu-MNP**

**10**^**2**^** × [Au]**_**0**_	**10**^**4**^** × [4-NP]**	**10**^**2**^** × *k*(min**^**-1**^**)**	**Correlation coefficient**
**(mg/mL)**	**(M)**		
0.221	1.5	13.79	0.9871
0.295	1.5	27.88	0.9966
0.369	1.5	36.9	0.9969
0.443	1.5	58.46	0.9987
0.295	0.5	73.87	0.9592
0.295	1.0	38.88	0.9840
0.295	2.0	22.78	0.9957

**Figure 8:**
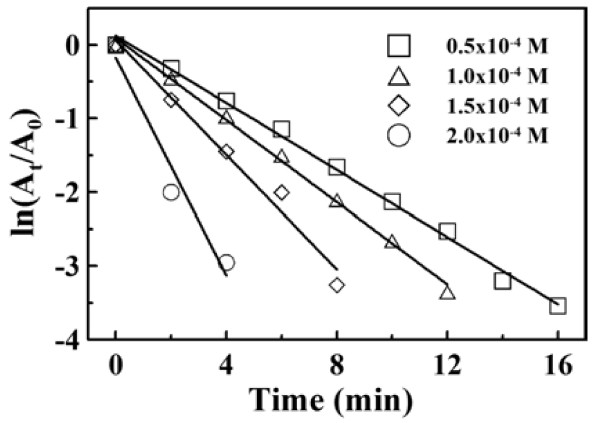
**Plots of ln(*A***_***t***_**/*A***_**0**_**) versus time for NaBH**_**4**_**-reduction of 4-NP at different initial concentrations of 4-NP.** [Au]_0_ = 0.295 × 10^−2^ mg/mL, [NaBH_4_]/[4-NP] = 100, 30°C.

The effect of initial 4-NP concentration on the reduction of 4-NP with NaBH_4_ at a fixed amount of GAAu-MNP was shown in Figure 8. It was obvious that all the ln(*A*_*t*_*/A*_0_) values decreased linearly with time for different initial 4-NP concentrations. The corresponding pseudo-first-order rate constants and correlation coefficients were also listed in Table 1. It was found that the pseudo-first-order rate constants decreased with the increase in the initial 4-NP concentration. The possible mechanism was further discussed as below.

It is known that the rate of electron transfer at the metal surface can be affected by the diffusion of 4-NP to the metal surface, interfacial electron transfer, and the diffusion of 4-AP away from the surface [15]. The observed rate constant can be expressed as follows: [15,47]

(1) $$1/k_{\mathrm{obs}}=\left(1/4\pi R^{2}\right)\left[\left(1/k_{\mathrm{et}}\right)+\left(R/D\right)\right]\text{,}$$

where *R* is the radius of metal nanoparticles, *D* is the diffusion coefficient, and *k*_et_ is the rate constant for electron transfer. Because smaller metal particles possess higher redox potential value which may accelerate the electron transfer [48], it was assumed that the heterogeneous charge transfer was faster than the diffusion for the Au nanoparticles-catalyzed reduction of 4-NP with NaBH_4_ due to the small diameter of Au nanoparticles (approximately 2 nm). Thus, Equation 1 could be reduced to the Smoluchowski expression as follows:

(2) $$k_{\text{obs}}=4\pi DR\text{.}$$

The rate constant was proportional to the diffusion coefficient, but the diffusion coefficient was inversely proportional to the concentration of 4-NP [49]. So, the effect of initial 4-NP concentration could be explained by the Smoluchowski expression. Also, the NaBH_4_-reduction of 4-NP by the GAAu-MNP developed in this work could be recognized as diffusion-controlled. This was similar to those observed in the reduction of 4-NP by the dendrimer-encapsulated metal (Ah, Pt, and Pd) nanoparticles [15] and the chitosan-stabilized Au nanoparticles [3]. As for the diffusion-controlled mechanism, it could be attributed to the GA layer on the surface of Fe_3_O_4_ nanoparticles where Au nanoparticles were *in situ* synthesized and stabilized.

**Figure 9:**
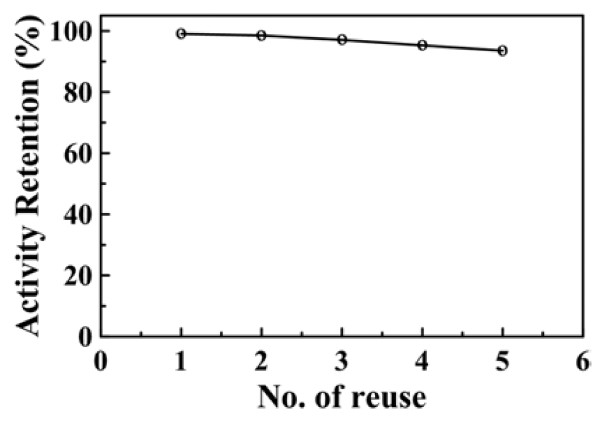
**Reusability of GAAu-MNP for the reduction of 4-NP with NaBH**_**4**_**.** [Au]_0_ = 0.295 × 10^−2^ mg/mL, [4-NP] = 1.5 × 10^−4^ M, [NaBH_4_] = 1.5 × 10^−2^ M, 30°C.

Figure 9 shows the reusability of the magnetically recoverable GAAu-MNP for the reduction of 4-NP with NaBH_4_. It was observed that the catalytic activity of GAAu-MNP for the NaBH_4_-reduction of 4-NP to 4-AP had no significant decrease after five cycles, suggesting that the GAAu-MNP was not deactivated or poisoned significantly during the reaction and recovery process. As compared to other metal nanoparticles, which were generally oxidized on the surface in the alkali solution and led to activity loss [47], the GAAu-MNP developed in this work was relatively stable and exhibited good catalytic ability.

According to the above findings, the green method for the synthesis of Au nanoparticles on the surface of magnetic nanoparticles has been developed successfully. Also, the resultant magnetically recoverable Au nanocatalyst was demonstrated to possess good catalytic property.

## Conclusions

A novel magnetically recoverable Au nanocatalyst was fabricated by *in situ* formation of Au nanoparticles on the surface of GA-modified Fe_3_O_4_ nanoparticles with GA as a reducing and stabilizing agent simultaneously. The resultant Au nanoparticles formed a 2 nm-thick layer on the surface of GA-modified Fe_3_O_4_ nanoparticles. Their catalytic ability was demonstrated by the study on the reduction of 4-NP to 4-AP with NaBH_4_. The reduction reaction followed the pseudo-first-order kinetics. The corresponding rate constants increased with the increasing amount of Au nanocatalyst but decreased while the initial 4-NP concentration increased, suggesting that the reaction was diffusion-controlled, owing to the presence of GA layer. Furthermore, the activity of magnetically recoverable Au nanocatalyst had no significant decrease after five recycles, revealing its good stability. Such a product is expected to be useful in the waste water treatment.

## Competing interests

The authors declare that they have no competing interests.

## Authors’ contributions

C-CW carried out the experiments and drafted the manuscript. D-HC guided the research and modified the manuscript. Both authors read and approved the final manuscript.

## Author’s information

D-HC (PhD) is a distinguished professor of Chemical Engineering Department at National Cheng Kung University (Taiwan). C-CW received his PhD in Chemical Engineering at National Cheng Kung University (Taiwan) in 2010 and now works as a researcher in Eternal Chemical Co., Ltd. (Taiwan).
